# Regulation of bacteria population behaviors by AI-2 “consumer cells” and “supplier cells”

**DOI:** 10.1186/s12866-017-1107-2

**Published:** 2017-09-19

**Authors:** Yufen Quan, Fankang Meng, Xinyu Ma, Xinhao Song, Xiao Liu, Weixia Gao, Yulei Dang, Yao Meng, Mingfeng Cao, Cunjiang Song

**Affiliations:** 1 Key Laboratory of Molecular Microbiology and Technology for Ministry of Education, Nankai University, Tianjin, 300071 China; 2 Department of Chemical and Biological Engineering, Iowa State University, Ames, IA 50011 USA

**Keywords:** Autorinducer-2, “Consumer cells”, “Supplier cells”, Biofilm

## Abstract

**Background:**

Autoinducer-2 (AI-2) is a universal signal molecule and enables an individual bacteria to communicate with each other and ultimately control behaviors of the population. Harnessing the character of AI-2, two kinds of AI-2 “controller cells” (“consumer cells” and “supplier cells”) were designed to “reprogram” the behaviors of entire population.

**Results:**

For the consumer cells, genes associated with the uptake and processing of AI-2, which includes LsrACDB, LsrFG, LsrK, were overexpressed in varying combinations. Four consumer cell strains were constructed: *Escherichia coli* MG1655 pLsrACDB (NK-C1), MG1655 pLsrACDBK (NK-C2), MG1655 pLsrACDBFG (NK-C3) and MG1655 pLsrACDBFGK (NK-C4). The key enzymes responsible for production of AI-2, LuxS and Mtn, were also overexpressed, yielding strains MG1655 pLuxS (NK-SU1), and MG1655 pLuxS-Mtn (NK-SU2). All the consumer cells could decrease the environmental AI-2 concentration. NK-C2 and NK-C4 were most effective in AI-2 uptake and inhibited biofilm formation. While suppliers can increase the environmental AI-2 concentration and NK-SU2 was most effective in supplying AI-2 and facilitated biofilm formation. Further, reporter strain, MG1655 pLGFP was constructed. The expression of green fluorescent protein (GFP) in reporter cells was initiated and guided by AI-2. Mixture of consumer cells and reporter cells suggest that consumer cells can decrease the AI-2 concentration. And the supplier cells were co-cultured with reporter cells, indicating that supplier cells can provide more AI-2 compared to the control.

**Conclusions:**

The consumer cells and supplier cells could be used to regulate environmental AI-2 concentration and the biofilm formation. They can also modulate the AI-2 concentration when they were co-cultured with reporter cells. It can be envisioned that this system will become useful tools in synthetic biology and researching new antimicrobials.

**Electronic supplementary material:**

The online version of this article (10.1186/s12866-017-1107-2) contains supplementary material, which is available to authorized users.

## Background

Microbial cells are widespread throughout the world. Intrinsic of bacterial plasticity to survive in diverse situations is their capacity to detect and respond to transient environments. As microorganisms usually share their environment with numerous other microorganisms, the communication amongst them plays a vital role in their survival. To view such systems as a whole “multicellular organism”, it is essential to develop effective strategies to regulate the behavior of the community [1]. Quorum sensing (QS) is a cell–cell communication that allows bacteria to sense one another and to regulate multicellular organism behaviors [2]. This system is regulated by signaling molecules known as autoinducers. Autoinducer-2 (AI-2) is a kind of biomolecule incorporating boron, which was first identified in the marine bacterium *Vibrio harveyi* [3, 4]. As a universal langue, AI-2 can be synthesized and recognized by both Gram-negative and Gram-positive bacteria [5, 6]. AI-2 could modulate a wide variety of cell-density dependent physiological activities including symbiosis, virulence, motility, antibiotic production and biofilm formation [7]. Based on the characteristics mentioned above, AI-2 is considered a kind of biosensor for regulating the activities dependent on quorum sensing.

QS networks dependent on AI-2 have been investigated in many bacteria such as *Helicobacter pylori, Bacillus cereus* and *Escherichia coli* K12 [8–10]. The mechanism of AI-2 uptake, processing and synthesis in *E.coli* has been well characterized. During active growth, LuxS produces AI-2, which is secreted into the extracellular space. AI-2 accumulates until it triggers the activation of the Lsr system in the receptor cells. LsrACDB, encoded by the genes of the *lsr* operon, are responsible for the internalization of AI-2 [11]. Other enzymes encoded by the *lsr* operon were involved in the regulation of gene expression and further intracellular metabolic degradation of AI-2 [12, 13].

**Fig. 1:**
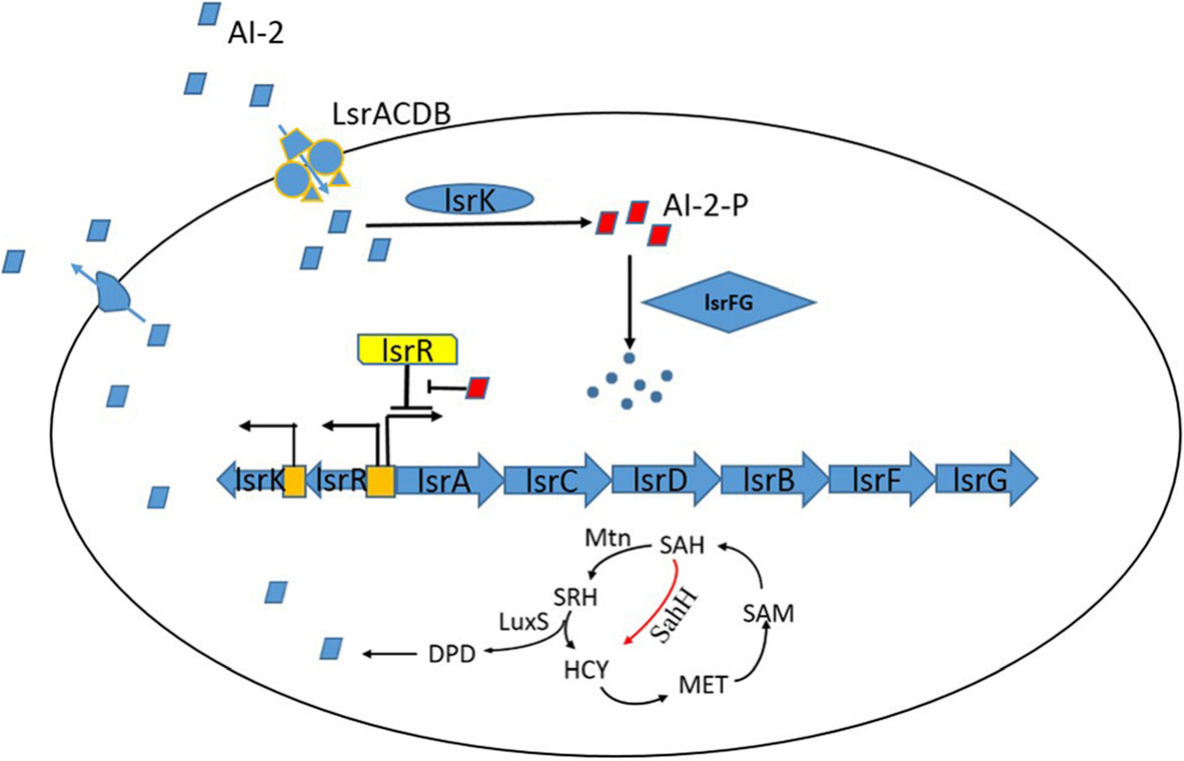
The uptake and synthesis of AI-2 in *E. coli*. AI-2 was transported into the cell by LsrACDB. LsrK was involved in the following phosphorylation of AI-2 signal molecule. When AI-2-P was accumulated to a specific value, the repression of *lsr* operon caused by LsrR was relieved. And then, the AI-2-P was degraded by LsrFG. AI-2 is the byproducts of the activated methyl cycle (AMC) and the process of AI-2 synthesis were depicted. Moreover, there is another pathway directing the conversion from SAH to HCY to complete the AMC, i.e. one-step conversion by SAH hydrolase (SahH). However, there was no AI-2 produced through the second route

For the synthesis of AI-2, previous studies suggest that under different biological contexts, the pathways for the formation of AI-2 are different [14]. One of the pathway was related to nucleic acid precursor, adenosine. The adding of glucose can modulate the metabolic flux and AI-2 synthesis [15]. However this pathway has not been illuminated. The AI-2 signal molecule is also a by-product of the activated methyl cycle (AMC). The substrate for AI-2 synthesis catalyzed by LuxS is S-ribosylhomocysteine (SRH), which derives from the toxic intermediate S-adenosylhomocysteine (SAH), a product from S-adenosylmethionine (SAM) metabolism, an important and ubiquitous central metabolite of the cell [11, 16]. During the process of AMC, SAH is hydrolyzed to SRH by 5′-methylthioadenosine/S-adenosylhomocysteine nucleosidase, which is called Pfs in *E. coli* W3110 and Mtn in MG1655 (Fig. 1).

Much research has focused on the QS system. Overexpression of the Pfs and LuxS could be used to AI-2 synthesis in vitro [17]. Based on the QS system in *E. coli*, exogenous addition of AI-2 to the QS synthase mutants was used to direct the assembly of “quantized quorums,” microbial subpopulations [18]. Furthermore, four kinds of bacterial AI-2 consumers were constructed by overexpressing components responsible for AI-2 uptake (*lsrACDB*), phosphorylation (*lsrK*), and degradation (*lsrFG*) [19]. These consumer cells were defined as “controller cells”, which can be deployed to control environmental AI-2 concentration. It has been previously demonstrated that consumer cells with *lsrACDB* overexpression are capable of silencing gene expression mediated by QS and can modulate chemotaxis and biofilm formation.

**Fig. 2:**
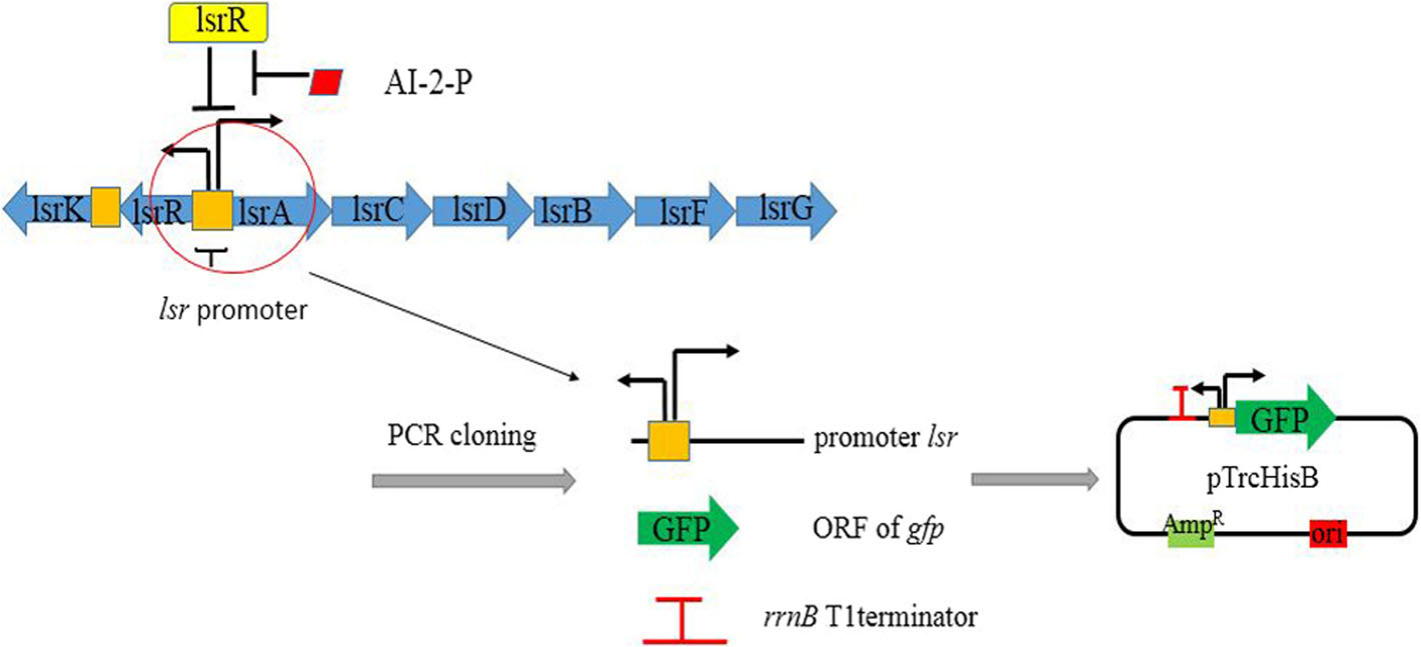
The plasmid used for the construction of reporter cells. The structure of *lsr* operon was depicted in the picture. The *lsr* is a bidirectional promoter. Three elements: *lsr* promoter, *gfp*, *rrnB* T1 terminator were ligated to pTrcHisB so that the plasmid pLGFP can respone to the intrecellular AI-2-P concentration

However, we thought that as the “controller cells”, they should not only include “consumer cell” capabilities, but also “supplier cell” functionality, capable of manipulating the AI-2 signaling molecule environmental concentration. In this study, the consumer cells were designed by overexpressing the components of *lsr* operon in varying combinations. Compared to the previous study, two new kinds of consumer strains were engineered by overexpressing *lsrACDBK* (NK-C2) and *lsrACDBEGK* (NK-C4). Both NK-C2 and NK-C4 have better AI-2 uptake capability than the consumer strains overexpressing *lsrACDB* and *lsrACDBFG.* In addition, supplier strains were constructed with *luxS* and/or *mtn* overexpression with the corresponding plasmids, pLuxS and pLuxS-Mtn. Since the controller cells were used to modulate the environmental AI-2 concentration, co-culture experiments were needed to characterize the function of controller cells. Then reporter cells were constructed. Briefly, pLGFP responsible for expression of GFP under the control of the *lsr* promoter (Fig. 2), was built and the expression of GFP can response to AI-2. MG1655 was transformed with pLGFP, resulting in the reporter strain (MG1655 pLGFP). The results indicated that supplier cells and consumer cells could modulate the environmental concentration of AI-2. With regards to biofilm effects, consumer cells could inhibit biofilm formation whereas supplier cells facilitate its formation. Besides that the supplier cells can activate GFP expression and the consumer cells silence GFP expression when they were co-cultured. These results suggest that supplier cells can provide more AI-2 in the environment while consumer cells can decrease the AI-2 concentration.

## Methods

### Strains and culture conditions

All strains and plasmids used in this study are listed in Table 1. *E. coli* MG 1655 was used as the basal strain. As needed, media were supplemented with antibiotics or IPTG with the following concentrations: 100 μg /mL ampicillin, 1 mM IPTG.

**Table 1:** Strains and plasmids used in this study

Strains and plasmids	Gene types and characteristics	Source
Strains		
*E. coli* MG 1655	*F-φ80 lac ZΔM15 Δ(lacZYA-arg F) U169 endA1 recA1 hsdR17*(*rk* ^*−*^ *,mk* ^*+*^) *supE44λ- thi − 1 gyrA96 relA1 phoA*	Transgene
*E. coli* NK-C1	MG1655 contains pLsrACDB	This study
*E. coli* NK-C2	MG1655 contains pLsrACDBK	This study
*E. coli* NK-C3	MG1655 contains pLsrACDBFG	This study
*E. coli* NK-C4	MG1655 contains pLsrACDBFGK	This study
*E. coli* SU1	MG1655 contains pLuxS	This study
*E. coli* SU2	MG1655 contains pLuxS-Mtn	This study
Plasmids		
pTrcHisB	Cloning vector Ap^r^	
pLsrACDB	pTrcHisB derivative, containing *lsrACDB*	This study
pLsrACDBFG	pTrcHisB derivative, containing *lsrACDB*, and *lsrFG*	This study
pLsrACDBK	pTrcHisB derivative, containing *lsrACDB* and *lsrK*	This study
plsrACDBFGK	pTrcHisB derivative, containing *lsrACDB*,*lsrFG,lsrK*	This study
pLuxS	pTrcHisB derivative, containing *luxS*	This study
pLuxS-Mtn pLGFP	pTrcHisB derivative, containing *luxS* and *mtn* pTrcHisB derivative, containing *lsr* promoter and *gfp*	This study This study

### Plasmid construction

The pTrcHisB was used as the plasmid backbone. The genes, *luxS, mtn, lsrACDB,* were PCR-amplified from the *E. coli* MG 1655 genome and ligated to pTrcHisB (*Kpn*I, *Eco*RI, *Bam*HI, *Sa*cI, *Bgl*II were used in this process) using ClonExpress II (Vazyme, China), to form three plasmids: pLuxS, pLuxS-Mtn, pLsrACDB. Then the *Pst*I and *Bst*BI digested *lsrK* segment was inserted into the same restriction sites of the pLsrACDB vector and transformed into *E.coli* K12, resulting in pLsrACDBK. The construction of plasmids pLsrACDBFG and pLsrACDBFGK was analogous to pLsrACDBK construction. For the construction of pLGFP, and the promoter sequence of *lsrR, lsr* and the opening reading frame (ORF) of green fluorescent protein (GFP) were PCR-amplified. This two fragments were ligated in pTrcHisB (*Bam*HI, *Sac*I were used in this process) using ClonExpress MultiS (Vazyme, China). Primers used in this study are listed in Additional file 1: Table S1. Procedures of cloning, DNA purification, and transformations were performed using standard protocols. All plasmids were confirmed by restriction enzyme digestion and DNA sequencing.

### Analysis of the gene expression

The levels of genes transcription and expression were analyzed using quantitative real-time PCR and Urea-PAGE. The *E. coli* MG 1655 pTrcHisB was used as the control. Strains harboring pLsrACDB, pLsrACDBK, pLsrACDBFG, pLsrACDBFGK, pLuxS, pLuxSMtn were cultured overnight. Strains were reinoculated and 1 mM IPTG was added into the medium when the strains reached the mid-log phase. After being induced for 4 h, the cells were collected for RNA extraction using RNApure Bacteria Kit (CWBIO, China). The target mRNAs were obtained by reverse-transcription and analyzed using the FastStart Universal SYBR Green Master (Rox). The expression of protein was evaluated by Urea-PAGE. Cell pellets were collected and resuspended with distilled water. Then cells were disrupted by a sonicator (300 W for 4 min with cycles of 5 s sonication followed by 10 s pause). Cell extracts were analyzed by Urea-PAGE following the standard protocol. Experiments were independently repeated at least three times, and the means and standard deviations were calculated.

### Analysis of AI-2 extracellular concentration

To validate the function of AI-2 consumer cells, extracellular AI-2 was first prepared. Compound A (Atomax Chemiscals Co. Ltd), defined as substance 11 in the previous study, was used for the supplement of 4, 5-dihydroxy-2, 3-pentanedione (DPD). The procedure of producing synthetic DPD was performed as the manuscript description [20]. First, compound A was dissolved in double-distilled water (ddH_2_O) and adjusted to pH 2 by H_2_SO_4_. After the reaction proceeded for 2 h, the resultant mixture was diluted with 0.1 M potassium phosphate buffer (pH = 7) to adjust the pH to 7.3. The obtained material, synthetic DPD, could be used without further purification in the *V. harveyi* bioassay, and displayed an activity equal to that of enzymatically prepared DPD. The synthetic DPD undergoes spontaneous rearrangements to form AI-2.

The uptake rate of AI-2 was analyzed as previously reported [21]. AI-2 (40 μM) and IPTG (1 mM) were added into the culture during mid-logarithmic phase (when the optical density at 600 nm (OD_600 nm_) was about 0.4). An Alltech system controller (Alltech Associates Inc., USA) equipped with a C18 reverse-phase column and UV-visible detector was used for AI-2 analysis. Formic acid of 0.01% (*V*/V) and acetonitrile was used as the mobile phase at a flow rate of 1 mL/min. Absorbance at 240 nm was recorded and used to calculate AI-2 concentration. The relative concentration changes was used to assess uptake capability of consumer cells.

The resulting environmental AI-2 concentration of the supplier cells was measured using high performance liquid chromatography (HPLC). Briefly, Strains were cultured overnight and inoculated. Then IPTG was added into the cultures during mid-logarithmic phase (about 4 h after inoculated) and then the AI-2 concentration was detected. *E. coli* MG 1655 pTrcHisB was used as the control.

### Characterization of biofilm

The MG1655 pTrcHisB base strain, four strains of AI-2 consumer cells, and two strains of supplier cells were cultured individually overnight and diluted to OD_600 nm_ = 0.05. Each AI-2 consumer cell strain and supplier cell strain was co-cultured with MG1655 pTrcHisB at a 1:1 (*v*/v) ratio with a total volume of 200 μl [22]. MG1655 pTrcHisB was cultured as control. Strains were grown in polystyrene 96-well plates at 30 °C overnight without shaking. IPTG was added into the culture at an OD_600 nm_ 0.4. To quantify the biofilm mass, the cell suspension was discarded and the plate was washed 3 times with PBS buffer (0.1 M, pH = 7). The plate was then incubated at 60 °C with the lid off for 60 min. The biofilms were stained with 0.1% crystal violet for 15 min and the excess dye was washed with water. The remaining dye (staining the biofilms) was dissolved with 95% ethanol and incubated with shaking for 30 min, and the OD at 540 nm was measured. Experiments were independently repeated at least three times, and the means and standard deviations were calculated. A two-tailed unpaired Student’s t-test was performed between the groups.

### To validate the function of AI-2 “reporter cells”

The function of reporter cells (MG1655 pLGFP), were validated as following. MG1655 pLGFP were cultured overnight and inoculated into fresh medium for 2 h (OD 600 = 0.2). Exogenous AI-2 was added into the culture. The final concentration of AI-2 was 50 μM, 40 μM, 30 μM, 20 μM, 10 μM, 0 μM, respectively. The 96-well black plates which contains cell cultures were subjected to fluorescence intensity determination through EnSpire Multimode Plate Reader using the 530/60 nm filter.

### Environmental concentration of AI-2 in co-cultures

Strains were cultured and inoculated at 37 °C with shaking at 180 rpm. IPTG was added into cultures at mid-log growth phase (OD_600_ = 0.40–0.60). Each AI-2 consumer cell strain and supplier cell strain was co-cultured with MG1655 pLGFP at a 1:1 (*v*/v) ratio with a total volume of 200 μl. MG1655 pLGFP co-cultured with MG1655 pTrcHisB was used as control. Fluorescence intensity and OD_600_ were measured every two hours. Experiments were independently repeated at least three times, and the means and standard deviations were calculated. A two-tailed unpaired Student’s t-test was performed between the groups.

## Results

### Construction of AI-2 “consumer cells” and “supplier cells”

**Fig. 3:**
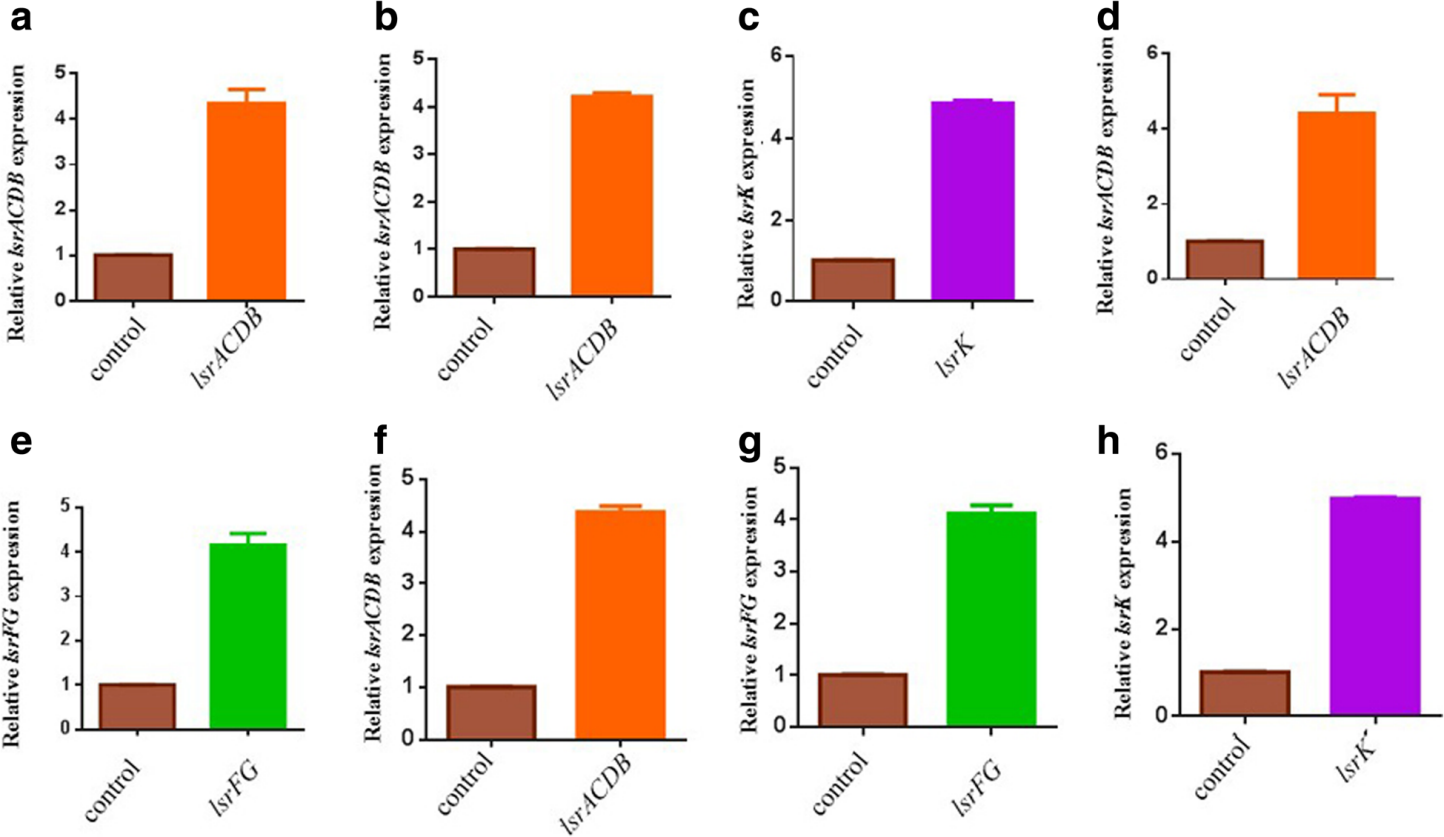
The quantitative real-time PCR of AI-2 “consumer cells”. **a**. The relative transcription level of *lsrACDB* in *E. coli* NK-C1. **b** and **c**. The relative transcription levels of *lsrACDB* and *lsrK* in *E. coli* NK-C2. **d** and **e**. The relative transcription levels of *lsrACDB* and *lsrFG* in *E. coli* NK-C3. **f**, **g** and **h**. The relative transcription levels of *lsrACDB*, *lsrFG* and *lsrK* in *E. coli* NK-C4. The relative transcription levels of lsrA and lsrF represented the transcription levels of lsrACDB and lsrFG,respectively. The strain containig pTrcHisB was defined as 1 in the expression level of corresponding genes

Two consumer cells were constructed to overexpress either *lsrACDBK*, or *lsrACDBFGK* and were named as *E. coli* NK-C2 (MG1655 pLsrACDBK) and *E. coli* NK-C4 (MG1655 pLsrACDBFGK). Another two consumer cells were also constructed, NK-C1 (MG1655 pLsrACDB) and *E. coli* NK-C3 (MG1655 pLsrACDBFG). To verify whether the consumer cells played the expected role, quantitative real-time PCR and Urea-PAGE were conducted (Fig. 3, Additional file 2: Figure S1a). The results confirmed that all the target genes in consumer cells were overexpressed.

**Fig. 4:**
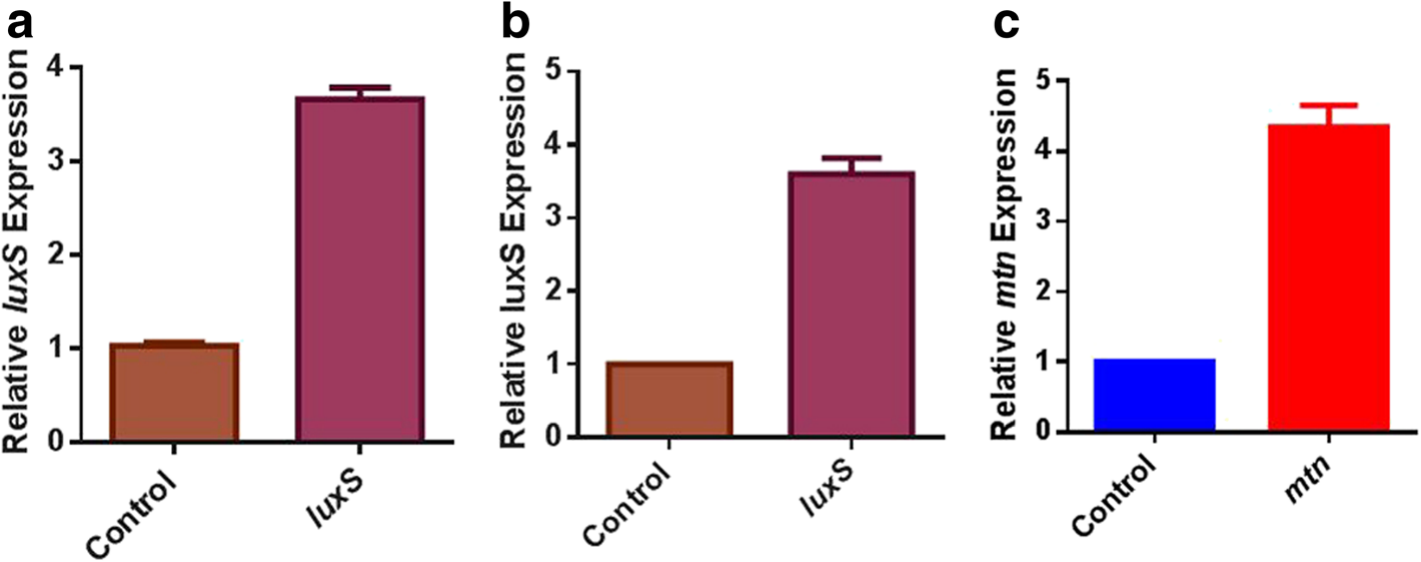
The quantitative real-time PCR of AI-2 “supplier cells”. **a**. The relative transcription level of *luxS* in *E. coli* NK-SU1. **b** and **c**. The relative transcription levels of *luxS* and *mtn* in *E. coli* NK-SU2. The strain containing pTrcHisB was defined as 1 in the expression level of corresponding genes

Along with the AI-2 consumer cells, supplier cells that could increase the environmental AI-2 concentration of were also constructed. In this study, the AI-2 synthesis genes *luxS* and *mtn* were cloned into pTrcHisB, yielding pTrcLuxS and pTrcLuxS-Mtn. The plasmids were transformed into *E. coli* MG1655, resulting in two supplier cell strains named *E. coli* NK-SU1 (MG1655 pLuxS) and NK-SU2 (MG1655 pLuxS-Mtn). The transcription and expression levels of *luxS* and *mtn* are summarized in Fig. 4 and Additional file 2: Figure S1b. In NK-SU1 and NK-SU2, *luxS* expression level was 3.5-fold higher than the control, whereas the *mtn* expression in NK-SU2 was around 4-fold higher than the control. The consumer cells and supplier cells were constructed by modulating the expression of genes associated with AI-2 synthesis and degradation. These modifications are listed in Additional file 3: Table S2.

### Environmental concentration of AI-2 controlled by “consumer cells” and “supplier cells”

**Fig. 5:**
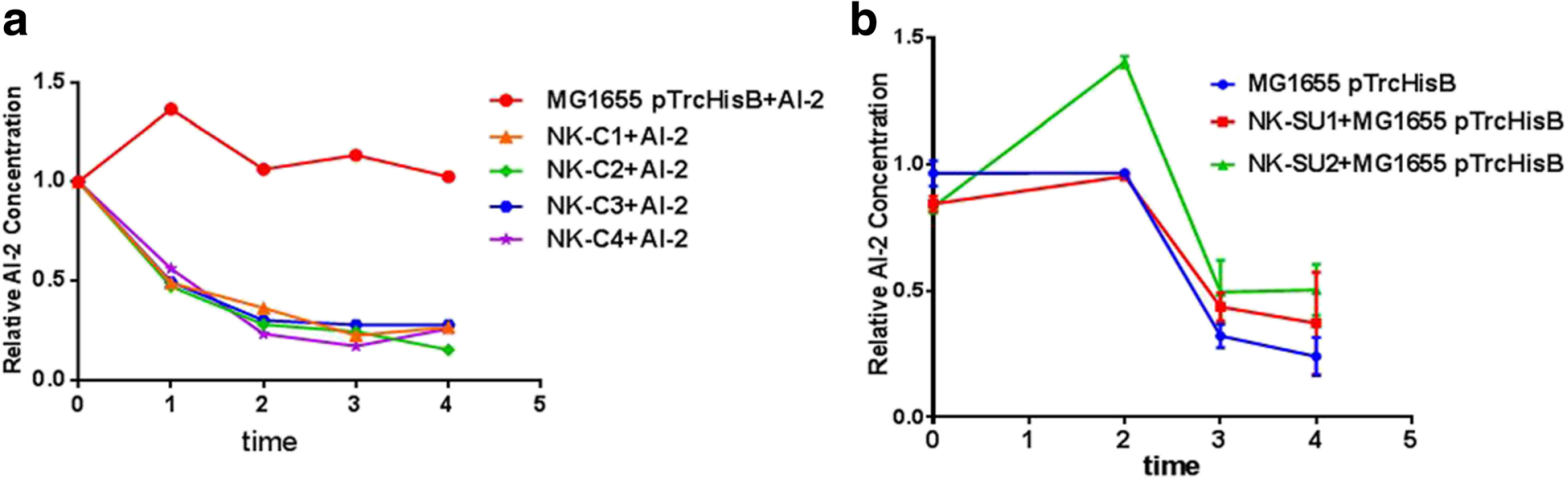
Effects of “consumer cells” and “supplier cells” on environmental AI-2 concentration. **a**. AI-2 uptake profiles of “consumer cells”. The relative environmental concentration of AI-2 of the control was defined as 1. **b**. AI-2 supply profiles of “AI-2 supplier cells”. The relative concentration of AI-2 was analyzed. The relative environmental concentration of AI-2 was defined as 1. The error bar indicated the standard deviation and each experiment was carried out by three replicates

It was verified that all the genes used for the construction of the six AI-2 consumer cell/supplier cell strains were expressed successfully. To further validate whether consumer cells could uptake AI-2, 40 μM exogenous AI-2 was added into the liquid culture. Then, the extracellular concentration of AI-2 was monitored by HPLC. Results showed that all consumer cells could successfully uptake the supplemented AI-2, compared to *E. coli* MG 1655 pTrcHisB. As shown in Fig. 5 a, environmental AI-2 concentration was significantly reduced after two hour induction with IPTG, which meant that consumer cells could “quench” the environmental AI-2 signal. Besides that the consumer cell strain NK-C2 and NK-C4, which overexpressed LsrK possessed effective AI-2 uptake capability, and the AI-2 uptake capability of NK-C4 was the greatest.

Additionally, environmental AI-2 concentration of supplier cells were also measured (Fig. 5 b). At the first 2 h, the AI-2 concentration of all strains increased. Then AI-2 concentration decreased significantly. During the last hour the decrease rate of AI-2 concentration became slower compared to that of the third hour. During the whole process, the AI-2 concentration of NK-SU2 were higher than NK-SU1 and MG1655 pTrcHisB. After induced for 4 h, the environmental concentration of AI-2 secreted by NK-SU1 and NK-SU2 resulted in 54% and 100% increases.

### Biofilm change caused by AI-2

**Fig. 6:**
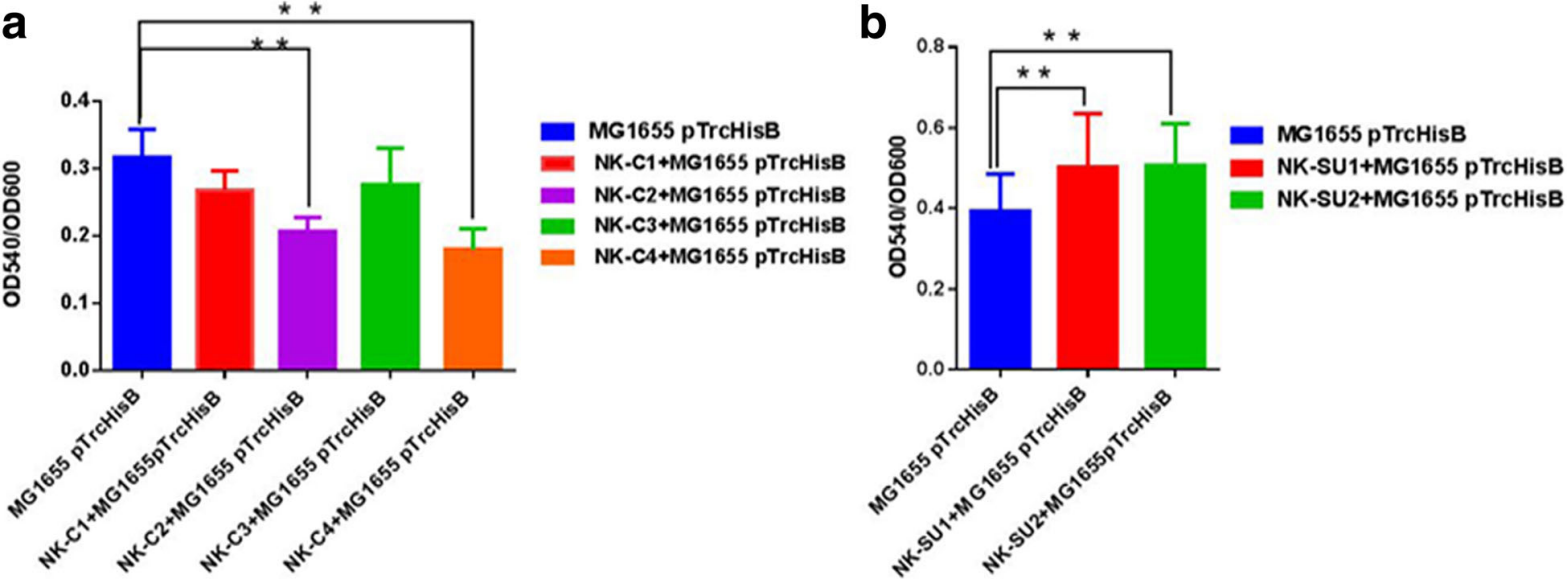
Effects of “consumer cells” and “supplier cells” on biofilm formation. **a**. Effects of “consumer cells” on biofilm formation. **b**. Effects of “supplier cells” on biofilm formation. The biofilm formation of MG1655 containing pTrcHisB was the control. The biofilm formation was characterized by OD_540 nm_/OD_600 nm_. The error bar indicated the standard deviation and each experiments was carried out by three replicates. A two-tailed unpaired Student’s t-test was performed between the groups and significant results indicated by the star. Two stars: *P* ≤ 0.05, three stars: *P* ≤ 0. 01

Nearly all cells make biofilms, which are formed in aquatic environments as the result of attachment of bacteria to submerged surfaces, to the air/liquid interface, and to each other [22, 23]. AI-2, being a universal signal molecule, can regulate many bacterial behaviors, including the formation of biofilms. Since the constructed consumer cells and supplier cells could regulate the environmental concentration of AI-2, they may also play a role in the formation of bacterial biofilm. Consumer cells and supplier cells were co-inoculated in parallel with MG1655 pTrcHisB to determine their effects on biofilm formation (Fig. 6) The biofilm thickness of MG1655 pTrcHisB co-cultured with NK-C1, NK-C2, NK-C3 or NK-C4 decreased by 15%, 34%, 12% and 43%, respectively, compared to the control. In contrast, the biofilm thickness of MG1655 pTrcHisB co-cultured with NK-SU1 or NK-SU2 increased by 27% and 28%, respectively, compared to the control (Fig. 6).

### Manipulation of “controller cells” in co-cultures

**Fig. 7:**
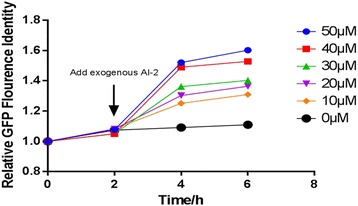
The response ability of reporter cells to exogenous AI-2. The strain containig pLGFP wihtout adding exougenous AI-2 was used as the control and its GFP flourence indensity was defined as 1. At the second hour, exogenous AI-2 were added into the culture at different concentration

The native QS regulon was re-engineered and AI-2 was used to guide high level expression of recombinant proteins in *E. coli* [15]. In this study, the native QS system was rewired to construct reporter cells that could response to AI-2. The AI-2 quorum sensing network was depicted in the Fig. 1. Promoter *lsr* can be repressed by LsrR, affecting the expression of *lsr* operon. This process can be derepressed by AI-2-P. The plasmid pLGFP involved in reporter cells construction was built (Fig. 2). The expression of GFP can be regulated by the environmental AI-2 concentration. The response ability to AI-2 of MG1655 pLGFP is shown in Fig. 7. After adding the exogenous AI-2, reporter cells can respond to variant concentration of AI-2 by emitting different fluorescence intensity. These results suggest that reporter cells can be used as a detector for the environmental AI-2 concentration.

**Fig. 8:**
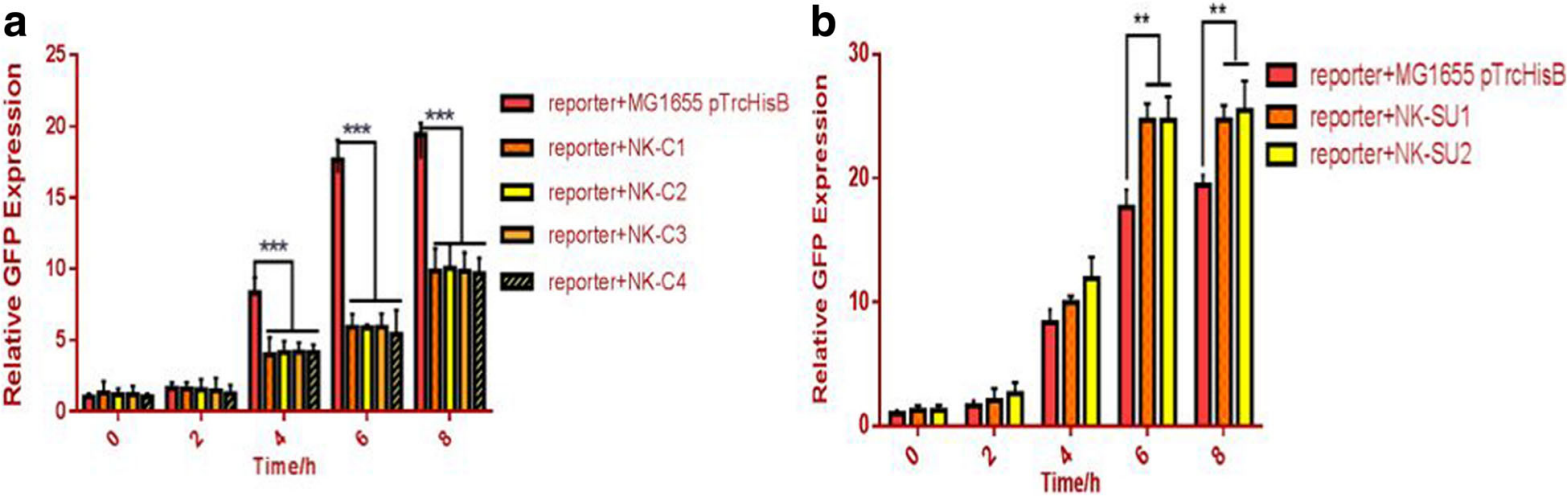
Manipulation of “controller cells” in co-cultures **a**. The regulation of GFP fluorescence by consumer cells. **b**. The regulation of GFP fluorescence by supplier cells. The strain containig pTrcHisB co-cultured with reporter cells was used as the control and its GFP flourence indensity at the beginning was defined as 1. The error bar indicated the standard deviation and each experiments was carried out by three replicates. A two-tailed unpaired Student’s t-test was performed between control and samples. And significant results indicated by the star. Two stars: *P* ≤ 0.05; three stars ≤0.01

AI-2 controller cells were co-cultured with reporter cells at the ratio of 1:1. Results showed that AI-2 consumer cells could significantly depress AI-2-inducible GFP fluorescence compared to the control group (Fig. 8 a), as expected. Also, at the 1:1 mixture of AI-2 reporter cells with AI-2 supplier cells, AI-2-inducible GFP fluorescence was significantly enhanced compared to the control group (Fig. 8 b). These suggest that our consumer cells do decrease the AI-2 concentration and supplier cells can increase the AI-2 concentration then regulate the GFP fluorescence. These results indicated that the controller cells could regulate the environmental AI-2 concentration.

## Discussion

Quorum-sensing networks enable bacterial cells to sense and communicate, which was needed for orchestrating population behavior. In this work, we constructed two kinds of controllers: AI-2 consumer cells and supplier cells. Since LsrACDB is the transporter of AI-2 and constitutes the rate-limiting step of AI-2 assimilation, these genes were overexpressed to construct numerous consumer cell strains. First, two consumer cell strains, similar with previous design [19], were constructed and named NK-C1 and NK-C3. Although extracellular concentration of AI-2 decreased, the AI-2 uptake of NK-C1 was not significantly different from NK-C3. One possible explanation is that LsrK is required for the phosphorylation of AI-2, which means AI-2 could be sequestrated in the cytoplasm [19], and AI-2-P is the optimum substrate for the LsrFG. It has been proved that LsrK can phosphorylate AI-2 in vitro, and LsrK-treated AI-2 can significantly reduce the native QS response of *E. coli* populations [24]. Therefore, two more consumer cell strains, NK-C2 and NK-C4, were constructed. Results indicated that the AI-2 uptake capability of NK-C2 was similar with that of NK-C4, with both strains possessing higher uptakes rates than NK-C1 and NK-C3. Moreover, consumer cells with LsrACDB and LsrK co-overexpression could absorb more AI-2 and prevent AI-2 from leaking out of the cell, which resulted in reduced environmental AI-2 concentrations. Since LsrFG is responsible for cellular degradation of AI-2-P, the effects of LsrFG on the environmental AI-2 were less notable. Thus, the uptake capability of NK-C2 was similar to that of NK-C4. In addition, both NK-C2 and NK-C4 possessed a strong capability to interfere with biofilm formation, coinciding with the uptake profile of extracellular AI-2. However, *luxS* has not been deleted. LuxS deletion could further improve AI-2 uptake capability.

In this study, *luxS* and *mtn* (also known as *mtn, pfs* or *yadA* in *E. coli* MG 1655) were manipulated in supplier cell design. Results indicated that NK-SU2 was more effective in increasing environmental AI-2 than that of NK-SU1. Previous studies with regards to enhancing AI-2 concentration were mostly focused on *luxS* overexpression [25]. In the AMC cycle, Mtn catalyzed the conversion of toxic intermediate SAH to SRH [26]. The co-expression of *mtn* and *luxS* could provide more substrate for AI-2 synthesis, resulting in higher AI-2 concentrations. And the AI-2 concentration increased in the logarithmic phase and dropped in the state-stage.

Biofilm formation of NK-C1 and NK-C2 was enhanced compared to the control, coinciding with the supply profile with AI-2. As biofilm formation occurs in 80% of human bacterial infections [22], and pathogens within biofilms have up to 1000 times higher antibiotic resistance compared to being present in a planktonic state, consumer cells have the potential to serve as next-generation antibiotic treatments due to their biofilm formation inhibition capabilities [27, 28]. Additionally, supplier cells can increase AI-2 concentration, which could increase bacterial stress resistance, benefiting the synthesis of probiotics products [25]. Thus, AI-2 consumer cells and supplier cells could be used to regulate different bacteria population behaviors.

## Conclusions

The two kinds of AI-2 controllers could be used as a tool to manipulate AI-2 concentrations which influences many cell density-dependent behaviors. Consumer cells can decrease AI-2 concentrations and repress biofilm formation. Supplier cells can increase AI-2 concentrations and facilitate biofilm formation. However, there are still some limitations in our present study. One is that consumer cells should be LuxS mutant strains to reduce the controller cells AI-2 concentration. Another is that our controller cells should be removed more convenient from the co-culture environment. Capsule or membrane can be used in the co-culture experiments.

## Additional files

Additional file 1: Table S1. Primers used in this study. (DOCX 13 kb)

Additional file 2: Figure S1. The expression of target genes. a. the expression of target genes in consumer cells. b. the expression of target genes in supplier cells. The masses of LsrA, LsrB, LsrC, LsrD, LsrF, LsrG, LsrK, LuxS, Mtn were 55.8kD, 36.6kD, 36.4kD, 34.4kD, 31.8kD, 57.45kD, 11.2kD, 19.4kD, 24.3kD, respectively. Compared to the control, there are three bands, 31kD, and 11.2kD in the Lane of pLsrACDBFG, which could indicate that the LsrF, LsrG were all overexpressed. The similar gel bands also indicated that LsrK, Mtn and LuxS were expressed in the target cells. Furthermore, the gel results were consistent with the qPCR results. (JPEG 294 kb)

Additional file 3: Table S2. The expression situation of corresponding genes in AI-2 “consumer cells” and “supplier cells”. (DOCX 12 kb)

## Abbreviations

AI-2: Autoinducer-2
AMC: activated methyl cycle
DPD: 4, 5-dihydroxy-2, 3-pentanedione
HCY: homocysteine
QS: quorum sensing
SAH: S-adenosyl homocysteine
SahH: SAH hydrolase
SAM: S-adenosylmethionine
SRH: S-ribosyl homocysteine
