# Supplementary material for: Regulation of bacteria population behaviors by AI-2 “consumer cells” and “supplier cells”
Source: BMC Microbiol. 2017 Sep 19;17:198. doi: 10.1186/s12866-017-1107-2 (PMC5605969; doi:10.1186/s12866-017-1107-2)
Supplement: Supplementary file 1 — Primers used in this study. (DOCX 13 kb) [file 12866_2017_1107_MOESM1_ESM.docx]

**Table 1s. Primers used in this study**

| **Primer name** | **Sequence** |
| --- | --- |
| *lsrACDBFG*1-F | CGACGATGACGATAAGGATCCATGCAAACGAGTGATACCCGC |
| *lsrACDBFG*1-R | TGTTTGGCGTTTCCGGCAGCGGTGCGGAGAGC |
| *lsrACDBFG*2-F | TGTTTGGCGTTTCCGCGATTG |
| *lsrACDBFG*2-R | GCACTCTCACACCACGTTGCATGGCGCGTTTC |
| *lsrACDBFG*3-F | GTGGTGTGAGAGTGCTGAC |
| *lsrACDBFG*3-R | ACCAGCTGCAGATCTCGAGCTCGTCACGGCATCAACCCATTGAAC |
| *lsrACDB*1-F | CGACGATGACGATAAGGATCCATGCAAACGAGTGATACCCGC |
| *lsrACDB*1-R | ACCGGAACCGCCGAGCAAACTAATGCCGCCCAGCAC |
| *lsrACDB*2-F | CTCGGCGGTTCCGGTGCGAT |
| *lsrACDB*2-R | ACCAGCTGCAGATCTCGAGCTCGTCAGAAATCGTATTTGCCG |
| *luxS*-F | ATGACGATAAGGATCCGAGCTCGATGCCGTTGTTAGATAGCTTC |
| *luxS*-R | GGACTCCCCCGGGGGACTAAATGTGCAGTTCCTGCAACTTC |
| *mtn*-F | TCCCCCGGGGGAGTCCTCTCCCGCGTGAGAAATAC |
| *mtn*-R | TATGGTACCAGCTGCAGATCTCTTAGCCATGTGCAAGTTTCTG |
| *luxS*-Fd | GGAATTCCCTAAATGTGCAGTTCCTGCAACTTC |
| *luxS*-Rv | GGGGTACCCCATGCCGTTGTTAGATAGCTTCACAG |
| *lsrK-*Fd | GGGGTACCCCATGGCTCGACTCTTTACC |
| *lsrK*-Rv | GGAATTCCCTATAACCCAGGCGCTTTCC |
| *lsrFG*-Fd | CGGGATCCCGATGGCAGATTTAGACGATATTAAAGATGG |
| *lsrFG*-Rv | GAAGATCTTCTCACGGCATCAACCCATTGAAC |
| *lsrK*-F | AACTGCAGAACCAATGCATTGGTTTACGGCTAGCTCAGTCCTAGGTATAGTGCTAGCAAAGAGGAGAAAATGGCTCGACTCTTTACC |
| *lsrK*-R | TTCGAACTATAACCCAGGCGCTTTCC |
| BK-JC-F | TTTCAGTGTATGTCGCGGATGC |
| GK-JC-F | TTGCGCTTCGATGTCTTACAGG |
| pTrc-JC-F | TGGGCACTCGACCGGAATTATC |
| pTrc-JC-R | GCTACTGCCGCCAGGCAAATTC |
