# Supplementary figures and images for: Regulation of bacteria population behaviors by AI-2 “consumer cells” and “supplier cells”
Source: BMC Microbiol. 2017 Sep 19;17:198. doi: 10.1186/s12866-017-1107-2 (PMC5605969; doi:10.1186/s12866-017-1107-2)

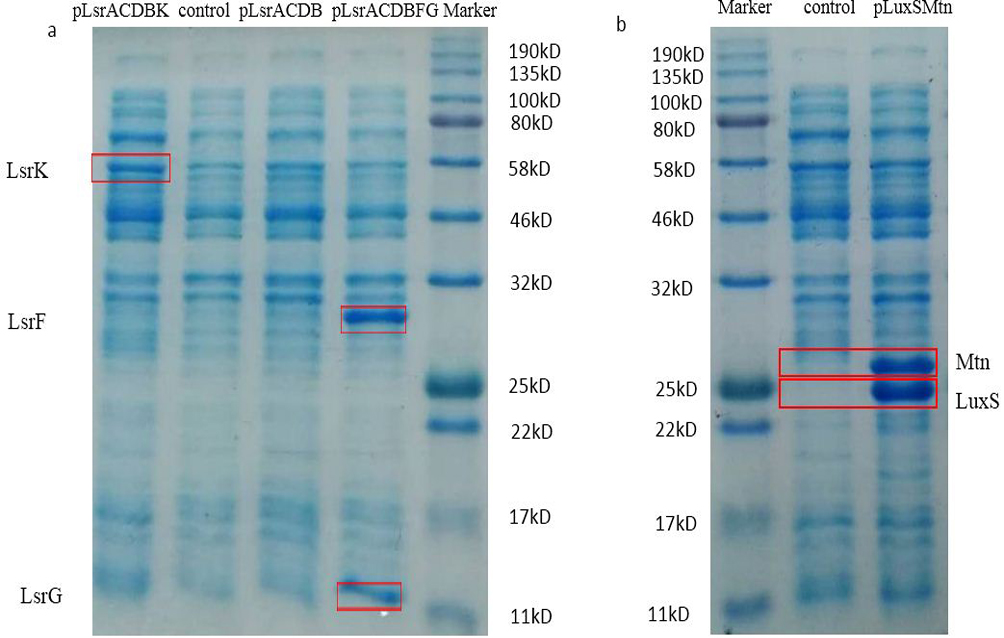

Supplement: Supplementary file 2 — The expression of target genes. a. the expression of target genes in consumer cells. b. the expression of target genes in supplier cells. The masses of LsrA, LsrB, LsrC, LsrD, LsrF, LsrG, LsrK, LuxS, Mtn were 55.8kD, 36.6kD, 36.4kD, 34.4kD, 31.8kD, 57.45kD, 11.2kD, 19.4kD, 24.3kD, respectively. Compared to the control, there are three bands, 31kD, and 11.2kD in the Lane of pLsrACDBFG, which could indicate that the LsrF, LsrG were all overexpressed. The similar gel bands also indicated that LsrK, Mtn and LuxS were expressed in the target cells. Furthermore, the gel results were consistent with the qPCR results. (JPEG 294 kb) [file 12866_2017_1107_MOESM2_ESM.jpg]
