# Supplementary material for: Regulation of bacteria population behaviors by AI-2 “consumer cells” and “supplier cells”
Source: BMC Microbiol. 2017 Sep 19;17:198. doi: 10.1186/s12866-017-1107-2 (PMC5605969; doi:10.1186/s12866-017-1107-2)
Supplement: Supplementary file 3 — The expression situation of corresponding genes in AI-2 “consumer cells” and “supplier cells”. (DOCX 12 kb) [file 12866_2017_1107_MOESM3_ESM.docx]

**Table 2s. The expression situation of corresponding genes in AI-2 “consumer cells” and “supplier cells”**

| AI-2 “consumer cells” | | | |
| --- | --- | --- | --- |
|  | *lsrACDB* | *lsrK* | *lsrFG* |
| *E. coli* MG1655 with pTrcHisB | Native^*^ | Native | Native |
| *E. coli* NK-C1 | Induced | Native | Native |
| *E. coli* NK-C2 | Induced | Induced | Native |
| *E. coli* NK-C3 | Induced | Native | Induced |
| *E. coli* NK-C4 | Induced | Induced | Induced |
| AI-2 “supplier cells” | | | |
|  | *luxS* | *mtn* |  |
| MG1655 TrcHisB | Native | Native |  |
| *E. coli* NK-SU1 | Induced^#^ | Native |  |
| *E. coli* NK-SU2 | Induced | Induced |  |

Native* indicates native production, while ‘Induced^#^’ indicates over-expression.
